# A bibliometric analysis of cerebral small vessel disease

**DOI:** 10.3389/fnagi.2024.1400844

**Published:** 2024-10-07

**Authors:** Xiaoxiao Yan, Yongyin Zhang, Ruqian He, Xiachan Chen, Mian Lin

**Affiliations:** ^1^Department of Neurology, The Third Affiliated Hospital of Wenzhou Medical University, Wenzhou, China; ^2^Department of Orthopedics, The Third Affiliated Hospital of Wenzhou Medical University, Wenzhou, China

**Keywords:** cerebral small vessel disease, CiteSpace, bibliometrics, VOSviewer, hot spots and frontiers

## Abstract

**Background:**

Cerebral small vessel disease (CSVD) is a significant contributor to both stroke and dementia. While numerous studies on CSVD have been published, herein, we have conducted a bibliometric examination of the literature on CSVD, revealing its hot spots and emerging patterns.

**Methods:**

We used the Web of Science Core Collection as our primary database and conducted a literature search from January 2008 to January 2023. CiteSpace, VOSviewer, online bibliometric platform, and R-bibliometrix were employed to conduct bibliometric analysis and network visualization, including the number of publications, countries, institutions, journals, citations, authors, references, and keywords.

**Results:**

A total of 4891 publications on CSVD were published in 790 journals by 19,066 authors at 3,862 institutions from 84 countries. The United States produced the most written works and had a significant impact in this field of study. The University of Edinburgh had the highest publication count overall. The journal with the most publications and co-citations was *Stroke*. Wardlaw, Joanna was the most prolific author and commonly cited in the field. The current areas of research interest revolved around “MRI segmentation” and “Enlarged perivascular spaces in the basal ganglia.”

**Conclusion:**

We conducted a bibliometric analysis to examine the advancements, focal points, and cutting-edge areas in the field of CSVD to reveal potential future research opportunities. Research on CSVD is currently rapidly advancing, with a consistent rise in publications on the topic since 2008. At the same time, we identified leading countries, institutions, and leading scholars in the field and analyzed journals and representative literature. Keyword co-occurrence analysis and burst graph emergence detection identified *MRI segmentation* and *Basal ganglia enlarged perivascular spaces* as the most recent areas of research interest.

## 1 Introduction

Cerebral small vessel disease (CSVD) refers to a collection of abnormal processes that impact the small blood vessels in the brain, including arteries, arterioles, venues, and capillaries (Pantoni, 2010). The primary features of CSVD include alterations to vessel walls leading to thickening, stiffening, and eventual occlusion of small vessels. The typical radiological and pathological signs of CSVD are lacunar infarcts, white matter lesions, cerebral microbleeds (CMBs), enlarged perivascular spaces (EPVS), and brain atrophy (Wu et al., 2022). Lacunar infarcts are caused by blockage of small arteries in the brain, leading to small subcortical infarcts. White matter lesions likely reflect ischemia and eventually lead to demyelination and axonal loss in the white matter tracts supplied by the diseased small vessels (Chen and Song, 2019). Cerebral microbleeds represent focal leakages of blood plasma and erythrocytes from diseased small vessels. EPVS signify enlarged fluid-filled canals surrounding small penetrating vessels and reflect altered interstitial fluid drainage (You et al., 2021). Brain atrophy results from the cumulative effects of ischemia and neuronal loss caused by CSVD. Furthermore, CSVD is a major cause of not only lacunar ischemic strokes but also intracerebral hemorrhage, which account for 20–30% of all ischemic strokes (Wardlaw et al., 2019) and cerebral hemorrhage (Francis et al., 2019). In addition to acute events, CSVD is associated with insidious neurological decline including cognitive, gait, and urinary impairment that includes the syndrome of vascular cognitive impairment (Horsburgh et al., 2018). With the global increase of the aging population, the burden of CSVD and resulting disabilities are expected to rise substantially in the coming decades (Arba et al., 2017). Therefore, to compile compelling evidence and provide valuable insights for the early diagnosis, prevention, and treatment of CSVD, we conducted the following bibliometric analysis.

Bibliometric analysis is the study of the structure, quantity, and impact of academic literature. This analysis can be conducted on individual researchers, specific fields of research, institutions, or countries. It involves the use of mathematical and statistical techniques to extract and analyze information from published academic literature. The aim of bibliometric analysis is to identify trends, patterns, and patterns of academic research. This analysis has important implications for the evaluation of research output, academic performance, and resource allocation (Chen and Song, 2019; Wu et al., 2022). Hence, we employed CiteSpace and VOSviewer to conduct a bibliometric analysis of the research on small vessel disease from 2008 to 2023 with the following objectives: (1) reveal the general information of CVSD over the past 15 years; (2) uncover the intractable problems and research hot spots related to CVSD; and (3) construct a knowledge graph in this field to provide valuable insights for future related research.

## 2 Methods

### 2.1 Data source and search strategy

**Figure 1:**
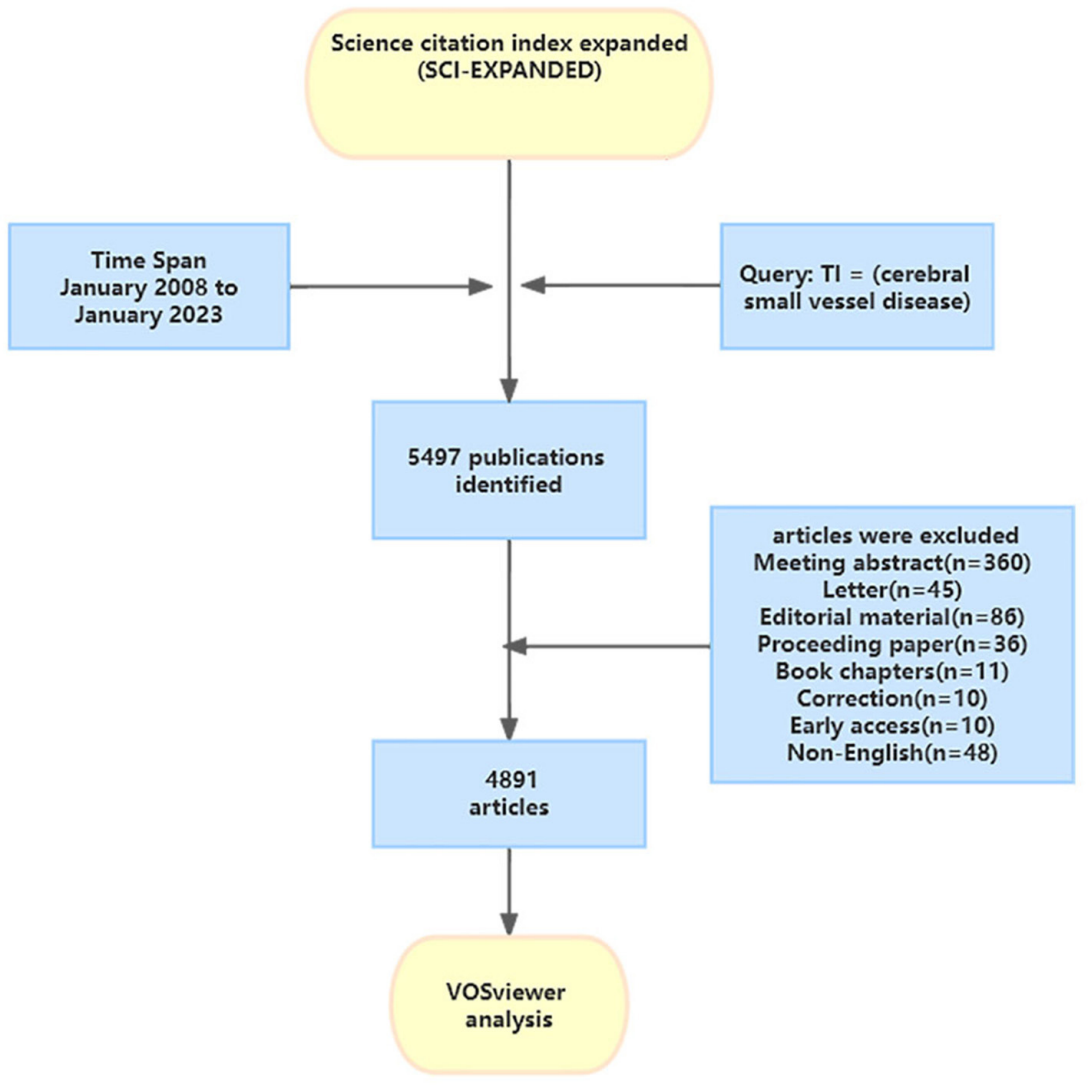
Flowchart for including and excluding literature studies.

A systematic search strategy was used to retrieve publications on CSVD from the Web of Science Core Collection (WoSCC) database between January 1, 2008 and January 1, 2023. Access to more than 21,000 journals in the fields of science, social sciences, and humanities is available through the WoSCC (You et al., 2021). We selected this database because of its multidisciplinary scope and citation indexing, which enables identification of the most impactful publications on CSVD. This database enabled us to identify publications related to the occurrence, causes, genetic factors, symptoms, identification, and treatment of CSVD. WoSCC is a recognized online database considered most suitable for bibliometric analysis (You et al., 2021). The search strategy was as follows: TI = (cerebral small vessel disease). The retrieval time was from January 2008 to January 2023. We limited the document types to “article” and “review.” Only papers published in English were searched. Because the set time span does not include the literature for the 11 months after January 2023, the data for 2023 is not complete, and the research analysis does not represent the whole year. The flowchart for the selection of publications is shown in Figure 1.

### 2.2 Data extraction and analysis

Complete data and referenced sources from all files in the WoSCC were obtained in either txt or BibTeX form and subsequently transferred to CiteSpace 6.1.R3, 64-bit (Drexel University, Philadelphia, PA, USA); VOSviewer 1.6.18 (Leiden University, The Netherlands); or R (Version 4.0.2), per the specific software needed for analyzing and displaying the information. CiteSpace was developed by Professor Chaomei Chen for visual analysis of bibliometrics. By analyzing a vast amount of reference data in a specific research area using co-occurrence and co-citation techniques, the CiteSpace software can provide an objective and quantitative analysis as well as predict future research frontiers and trends (Chen and Song, 2019). In this research, CiteSpace was used to analyze the dual-map overlap of journals and the cluster view and burst detection of cited literature. The parameters were set as follows: time span (2008–2023), years per slice (1), pruning (Minimum Spanning Tree and Pruning Sliced Networks), selection criteria (top *N* = 50), and others followed the default. VOSviewer 1.6.17 was used to identify productive co-cited authors, keywords, and related knowledge maps. The bibliometrics packages in R were used to analyze the trends of annual publications and the citations of the publications. The URL https://bibliometric.com/was used to analyze the changing trend of the annual publication quantity in the top 10 countries and the geographic distribution map of different countries.

## 3 Results

### 3.1 The annual growth trend of publications

**Figure 2:**
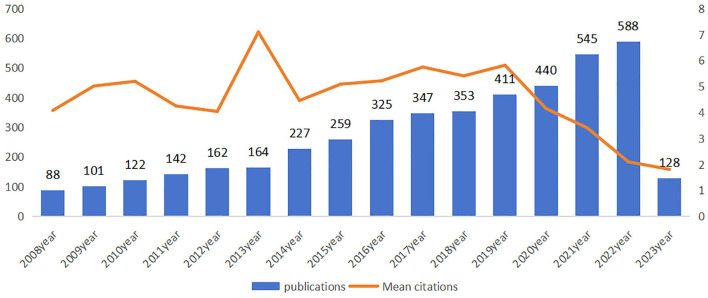
Number of annual publications and mean citation relating to CVSD from 2008 to 2023.

A total of 4891 publications on CVSD were retrieved from the WoSCC, including 4,056 articles and 835 reviews. The papers were written by 19,066 authors from 3,862 organizations across 84 countries, published in 790 journals, and cited 103,885 references from 8,783 journals. Dynamic changes on the number of publications during the past decade reflected the overall development trend in the field, with an average annual growth rate of 2.53%. As shown in Figure 2, the output of publications grew steadily from 2008 to 2022, especially in the last 2 years, although the average number of citations remained low. The average citation frequency of each article was 31.63, and the mean citation reached a peak in 2013, with a total of 164 articles and 7.1 mean citation, indicating that publications in 2013 might have contained groundbreaking or influential research findings.

### 3.2 Distribution of countries and institutions

The top 10 most productive countries and institutions in the CVSD field are presented in Table 1, including their publication count (NP), total citations (NC), and the average number of citations (AC). The United States (USA) led in publications with 1,182 papers (24.16%) and 52,342 citations, followed by China with 973 papers (19.89%) and the Netherlands with 563 papers (11.51%). It is important to mention that China's NC and AC were lower than the other top 10 productive countries, despite China being ranked second in NP.

**Table 1:** Summary of the leading 10 nations and organizations.

**Rank**	**Country**	**Number of publications**	**Number of citations**	**Average citation**	**Rank**	**Institution**	**Number of publications**	**Number of citations**	**Average citation**	**Country**
1	USA	1,182	52,342	44.28	1	University of Edinburgh	221	13,906	62.92	England
2	China	973	18,686	19.20	2	Harvard Medical School	195	5,115	26.23	USA
3	Netherlands	563	31,217	55.45	3	Massachusetts General Hospital	138	6,708	48.61	USA
4	England	532	26,731	50.25	4	University of Cambridge	138	5,183	37.56	England
5	Germany	408	20,031	49.10	5	Capital Medical University	132	1,407	10.66	China
6	France	330	18,186	55.11	6	Univ med ctr Utrecht	119	4,864	40.87	Netherlands
7	Japan	314	7,360	23.44	7	Radboud University Nijmegen	116	4,335	37.37	Netherlands
8	Italy	264	13,280	50.30	8	Leiden University	115	4,526	39.36	Netherlands
9	South Korea	253	6,125	24.21	9	Maastricht University	94	3,727	39.65	Netherlands
10	Canada	252	14,555	57.76	10	Fudan University	92	1,084	11.78	China

**Figure 3:**
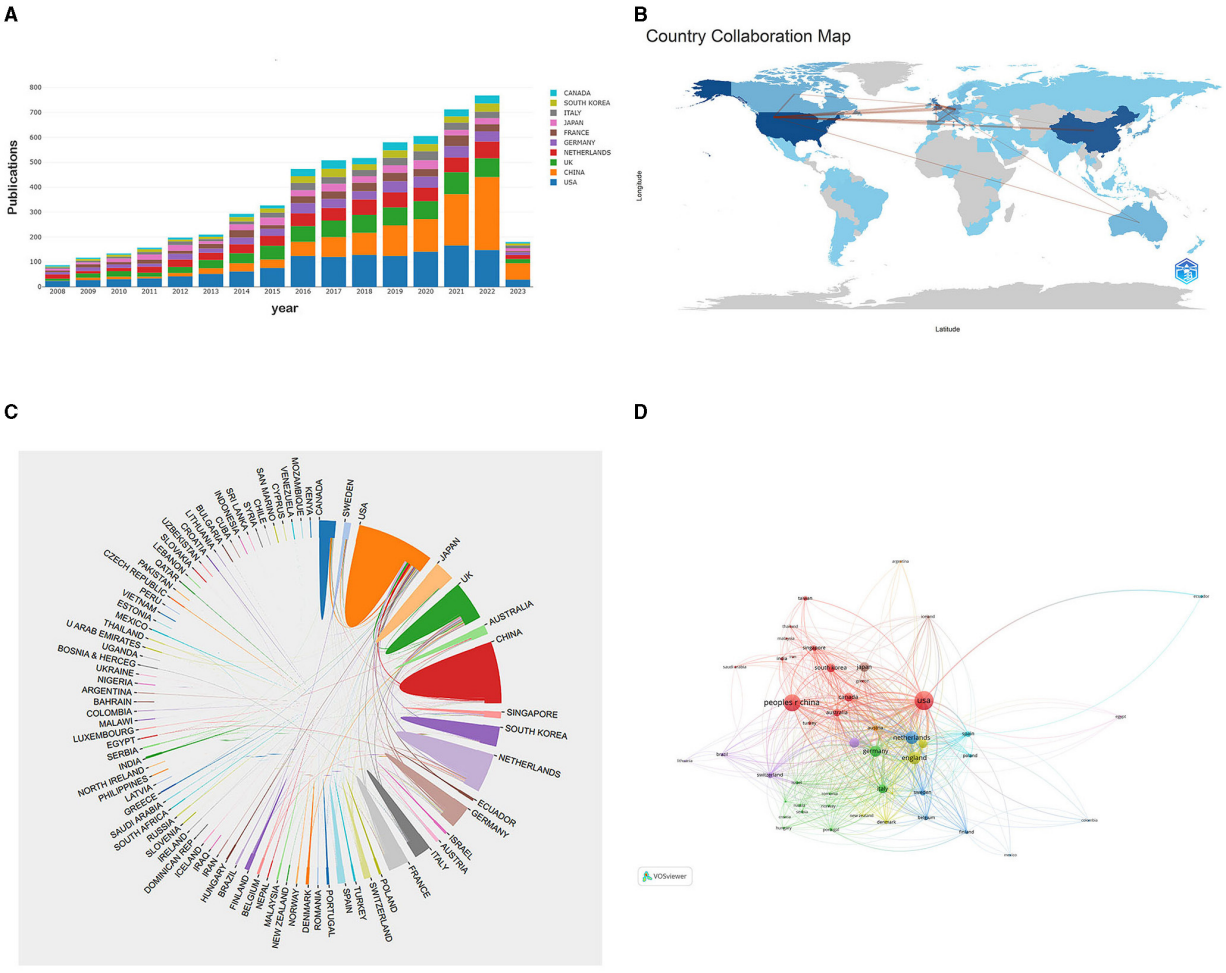
**(A)** The changing trend of the annual publication quantity in the top 10 countries from 2008 to 2023. **(B)** Geographic distribution map of different countries in the CVSD field. **(C)** The cross-country collaborations visualization map. **(D)** Network visualization showing the relationship between countries.

**Figure 4:**
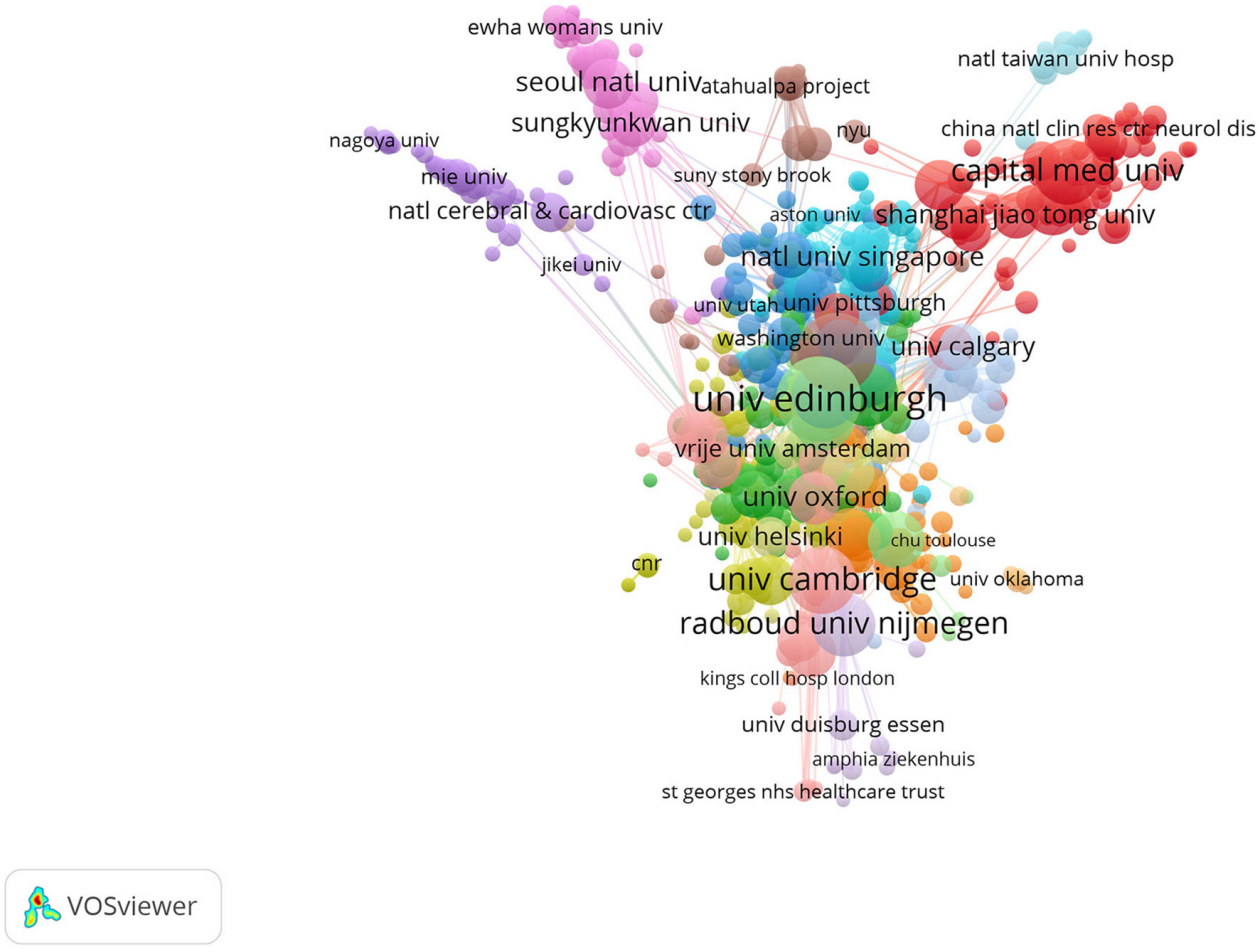
The network visualization of the citation analysis of the institutions.

In Figure 3A, the yearly publication count of the top 10 most productive nations is presented, indicating a rapid increase in publications related to CVSD. We utilized the Biblioshiny software to examine data and create visual representations to investigate international collaboration. The complexity of cooperation among various nations is illustrated in Figure 3B. As shown in Figure 3C, the international collaboration map among countries indicates that the USA collaborated most closely with China and England. VOSviewer analyzed the citation relationship between countries (Figure 3D). Countries that had at least 30 publications were considered. Out of the 48 countries and regions that reached this level, the leading five with the highest Total Link Strength (TLS) were the United States (TLS = 1,395), United Kingdom (TLS = 1,028), The Netherlands (TLS = 895), Germany (TLS = 888), and France (TLS = 728). The most influential institutions are outlined in Table 1. The institution with the highest NP and AC values was the University of Edinburgh (NP = 221). VOSviewer was used to analyze the citation connections among institutions (Figure 4). Institutions with at least 25 citation connections were considered for inclusion. Out of the 115 that qualified, the top five institutions with the highest TLS scores were the Massachusetts General Hospital (TLS = 54), Harvard Medical School (TLS = 44), University of Edinburgh (TLS = 34), University of Cambridge (TLS = 25), and Capital Medical University (TLS = 3).

### 3.3 Authors and co-cited authors

**Figure 5:**
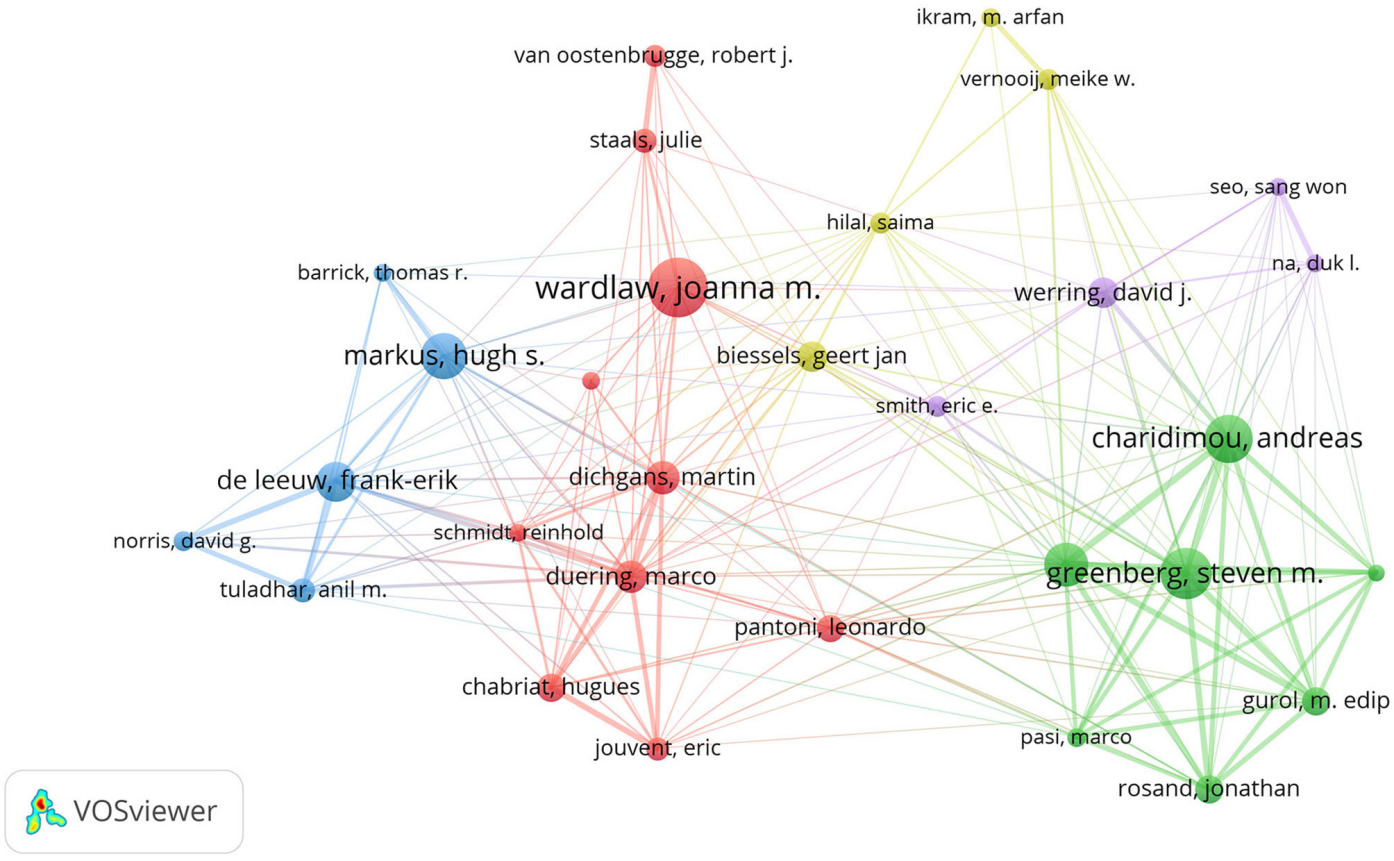
The network visualization of the co-authorship analysis of the authors.

Table 2 displays the top 10 authors and co-cited authors who have made significant contributions to the field of CVSD. Wardlaw, Joanna was the most productive author, with 116 articles and 9,178 citations. The most co-cited author was also Wardlaw, Joanna, with a TLS of 10,176. A minimum of 30 publications per author was required, and 34 authors were chosen for co-authorship analysis using VOSviewer. According to the co-authorship analysis (Figure 5), the authors were categorized into five different clusters. The largest group of authors, with 10 individuals, was the red cluster with the most co-authorships.

**Table 2:** Top 10 authors and co-cited authors of CSVD publications.

**Rank**	**Author**	**Publications**	**Citations**	**Average citation**	**Co-citations author**	**Citations**	**Total link strength**
1	Wardlaw, Joanna M.	116	9,178	79.12	Wardlaw, Joanna M.	3,477	10,176
2	Greenberg, Steven M.	99	7,433	75.08	Pantoni, L	1,758	6,476
3	Charidimou, Andreas	94	4,676	49.74	Charidimou, A	1,527	7,032
4	Markus, Hugh S.	89	3,975	44.66	Fazekas, F	1,376	5,168
5	Viswanathan, Anand	86	5,453	63.41	Greenberg, SM	1,061	5,247
6	De Leeuw, Frank-Erik	77	3,355	43.57	Joutel, A	772	1,771
7	Dichgans, Martin	66	5,223	79.14	Smith, EE	659	3,634
8	Duering, Marco	64	2,463	38.48	Debette, S	618	3,035
9	Duering, Marco	60	3,531	58.85	Vermeer, SE	616	2,820
10	Biessels, Geert Jan	59	3,576	60.61	Schmidt, R	569	2,931

### 3.4 Journals and co-cited journals

**Figure 6:**
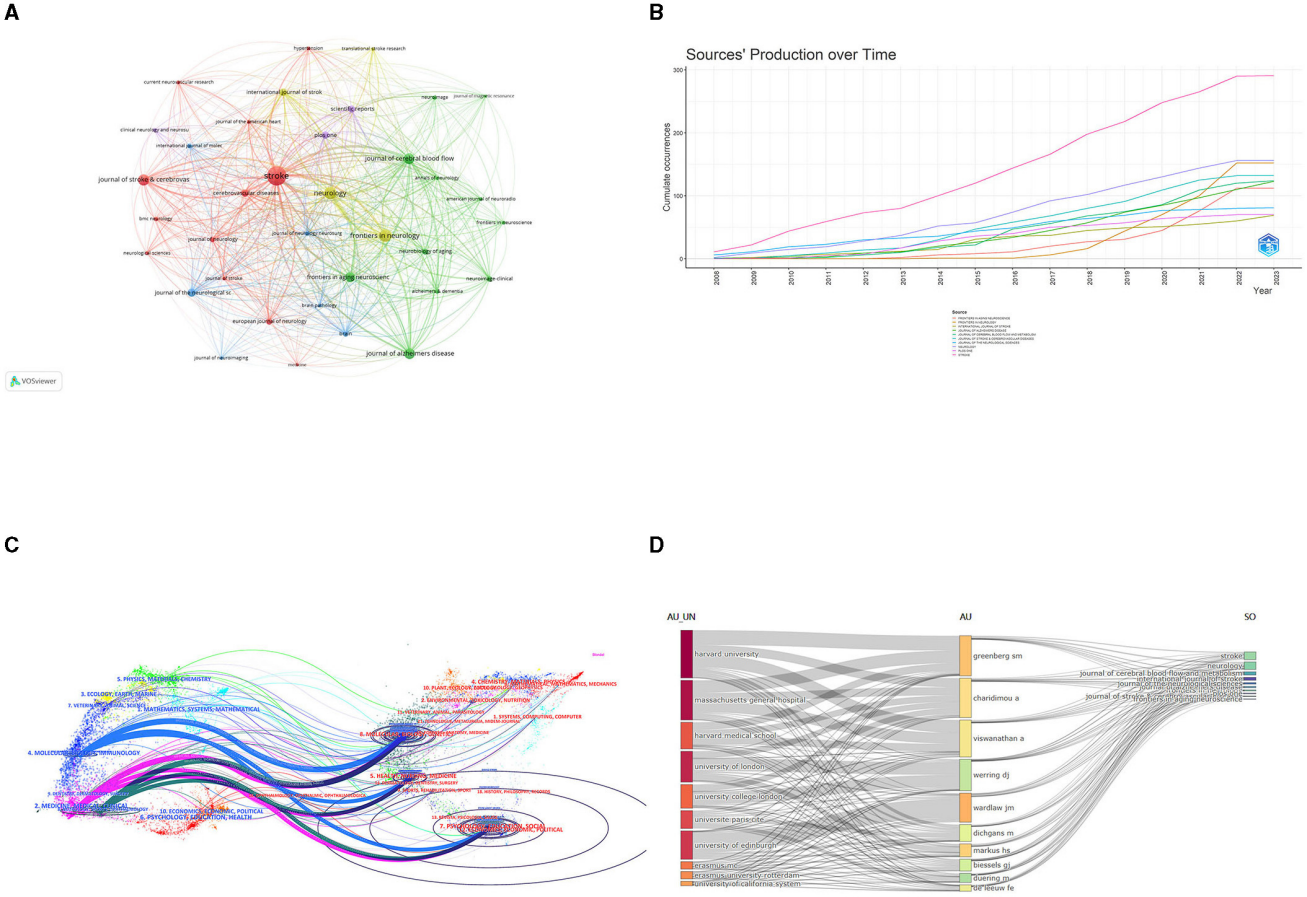
**(A)**The network visualization of the co-citation analysis of the journals. **(B)** The annual occurrence frequency of journal. **(C)** Dual-map overlay of journals on CVSD. **(D)** Three-field plots between institutions, authors, and publication sources. AU UN, Author University; AU, Author; SO, Sources.

*Stroke* published the most articles (291, 36.8%) out of the journals analyzed, indicating that it is a leading journal in publishing research related to CSVD, followed by *Neurology* (156, 19.4%). Among the top 10 journals, *Neurology* holds the highest impact factor (IF = 10.1). The influence of the top 10 most co-cited journals is determined by how frequently they are cited. Table 3 shows that *Stroke* had the highest number of citations (23,217), suggesting its rigorous acceptance standards and commitment to publishing influential research that contributes to the advancement of knowledge and practice in the field. The second-most prolific journal was *Neurology*. We visually analyzed the collaboration of published journals using VOSviewer (Figure 6A). *Stroke, Neurology*, and the *Journal of Cerebral Blood Flow and Metabolism* had more times of co-citation and a greater influence than other journals. We also analyzed the change pattern in the annual occurrence frequency of journals (Figure 6B). The number of publications in *Stroke* has always been leading and is gradually widening the gap with other journals. The number of publications in *Frontiers in Neurology* increased rapidly from 2018 to 2022. In Figure 6C, the dual-map overlay of journal publishing research is presented. Citing journals on the left and cited journals on the right, the curve is the citation line, which completely shows the context of the citation. The more papers the journal publishes, the longer the vertical axis of the CVSD; the more authors they are, the longer the horizontal axis of the CVSD (Figure 6C). Three main citation paths were identified. The blue paths indicate that the studies published in Molecular/Biology/Genetics journals and Health/Nursing/Medicine journals are usually cited in the studies published in Molecular/Biology/Immunology journals. The pink paths represent that studies published in the Molecular/Biology/Genetics/Health/Nursing/Medicine/ Psychology/Education/Social journals are typically cited in the studies published in Medicine/Medical/Clinical journals. The green paths represent that the studies published in Molecular/Biology/Genetic/Health/Nursing/Medicine/ Psychology/Education/Social journals are usually cited in the studies published in Neurology/Sports/ Ophthalmology journals.

**Table 3:** Top 10 journals and co-cited journals of CSVD publications.

**Rank**	**Journal**	**Publications**	**IF (Journal Citation Reports, 2022)**	**Co-citations journal**	**Citations**	**IF (Journal Citation Reports, 2022)**
1	Stroke	291	8.4	Stroke	23,217	8.4
2	Neurology	156	10.1	Neurology	16,194	10.1
3	Frontiers in Neurology	152	3.4	Lancet Neurol	7,570	48
4	Journal of Stroke & Cerebrovascular Diseases	132	2.5	Journal of Cerebral Blood Flow and Metabolism	4,401	1.6
5	Journal of Cerebral Blood Flow and Metabolism	124	6.3	Brainj Cerebr Blood F Met	4,288	0.5
6	Journal of Alzheimer's Disease	123	4	Ann Neurol	4,142	4.7
7	Frontiers in Aging Neuroscience	112	4.8	J Neurol Neurosur Ps	3,964	1.6
8	Journal of the Neurological Sciences	81	4.4	Neuroimage	3,865	5.7
9	PLOS One	70	3.7	Neurobiol Aging	3,024	5.9
10	International Journal of Stroke	69	6.7	Cerebrovascular Disease	2,843	1.9

The co-occurrence relationships between institutions, authors, and publication sources are presented in a three-field plot (Figure 6D). The size of each node represented its frequency or importance, while the connections between nodes represented co-occurrence relationships between two indicators, with the thickness of the lines indicating the frequency or strength of the co-occurrence. The most co-occurrence relationship existed between the institutions “Harvard university” is predominantly associated with the author “Greenberg, Steven M.” and the Journal of stroke and neurology. Notably, certain authors exhibited strong co-occurrence with specific institutions; for example, “Charidimou, Andreas” and “Viswanathan, Anand” were closely linked with Harvard University. With respect to journals, *Stroke* and *Neurology* contributed largely to all these authors' publications. These observations shed light on influential authors and institutions in this research domain and provide insights into potential future research directions.

### 3.5 Co-cited references and references burst

**Figure 7:**
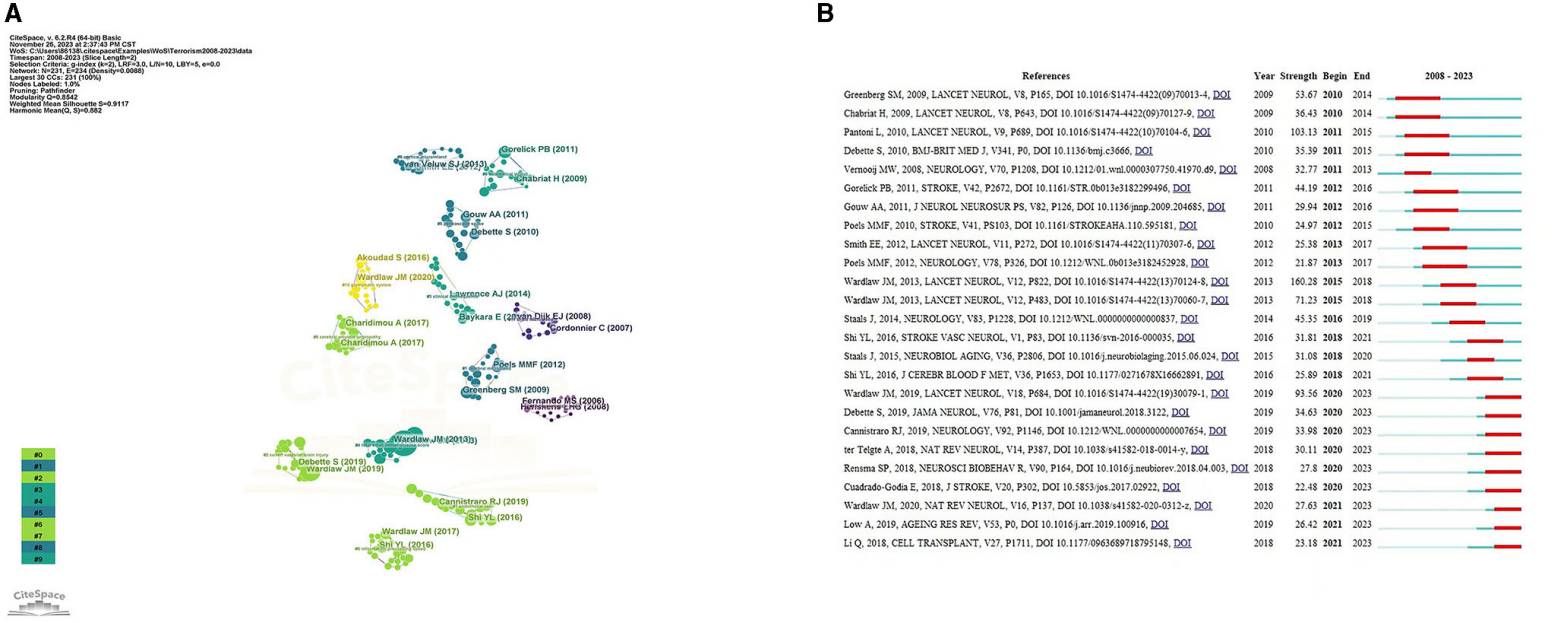
**(A)** References analysis to CVSD. **(B)** Top 25 References with the strongest citation bursts.

The top 10 documents that were cited the most are listed in Table 4. The paper titled “Neuroimaging standards for studying small vessel disease and its impact on aging and neurodegeneration” by Wardlaw et al. (2013a) is the most frequently referenced, with 3,276 citations. CiteSpace's clustering function was utilized to categorize co-cited references (Figure 7A), revealing the primary author and top 10 most-cited references. The literature that is cited most often and has the most significant bursts of citations is seen as the foundation for upcoming innovative research. This is illustrated in Figure 7B, which displays the top 25 bursts of citations in chronological order. The blue line indicates the observed time interval from 2008 to 2023, and the red color represents the duration of the burst, thus illustrating research hot spots and durations. The above paper authored by Wardlaw, Joanna achieved the highest citation strength for outbreaks (strength = 160.28). Nine articles focusing on the mechanism, pathology, and clinical features of CSVD were published starting in 2020.

**Table 4:** Top 10 co-cited references related to CVSD.

**Paper**	**DOI**	**Total citations**	**TC per year**	**Normalized TC**
Wardlaw et al. (2013a), Lancet Neurol	10.1016/S1474-4422(13)70124-8	3,276	297.82	41.97
Pantoni (2010), Lancet Neurol	10.1016/S1474-4422(10)70104-6	2,086	149	28.67
Greenberg et al. (2009), Lancet Neurol	10.1016/S1474-4422(09)70013-4	1,255	83.67	16.68
Iadecola (2013), Neuron	10.1016/j.neuron.2013.10.008	1,108	100.73	14.2
Wardlaw et al. (2013b), Lancet Neurol-A	10.1016/S1474-4422(13)70060-7	1,027	93.36	13.16
Sweeney et al. (2019), Physiol Rev	10.1152/physrev.00050.2017	939	187.8	32.26
Nation et al. (2019), Nat Med	10.1038/s41591-018-0297-y	751	150.2	25.8
Prins and Scheltens (2015), Nat Rev Neurol	10.1038/nrneurol.2015.10	656	72.89	14.32
Wardlaw et al. (2019), Lancet Neurol	10.1016/S1474-4422(19)30079-1	641	128.2	22.02
Mansour et al. (2018), Nat Biotechnology	10.1038/nbt.4127	621	103.5	19.13

### 3.6 Key topics of research hot-spots

**Figure 8:**
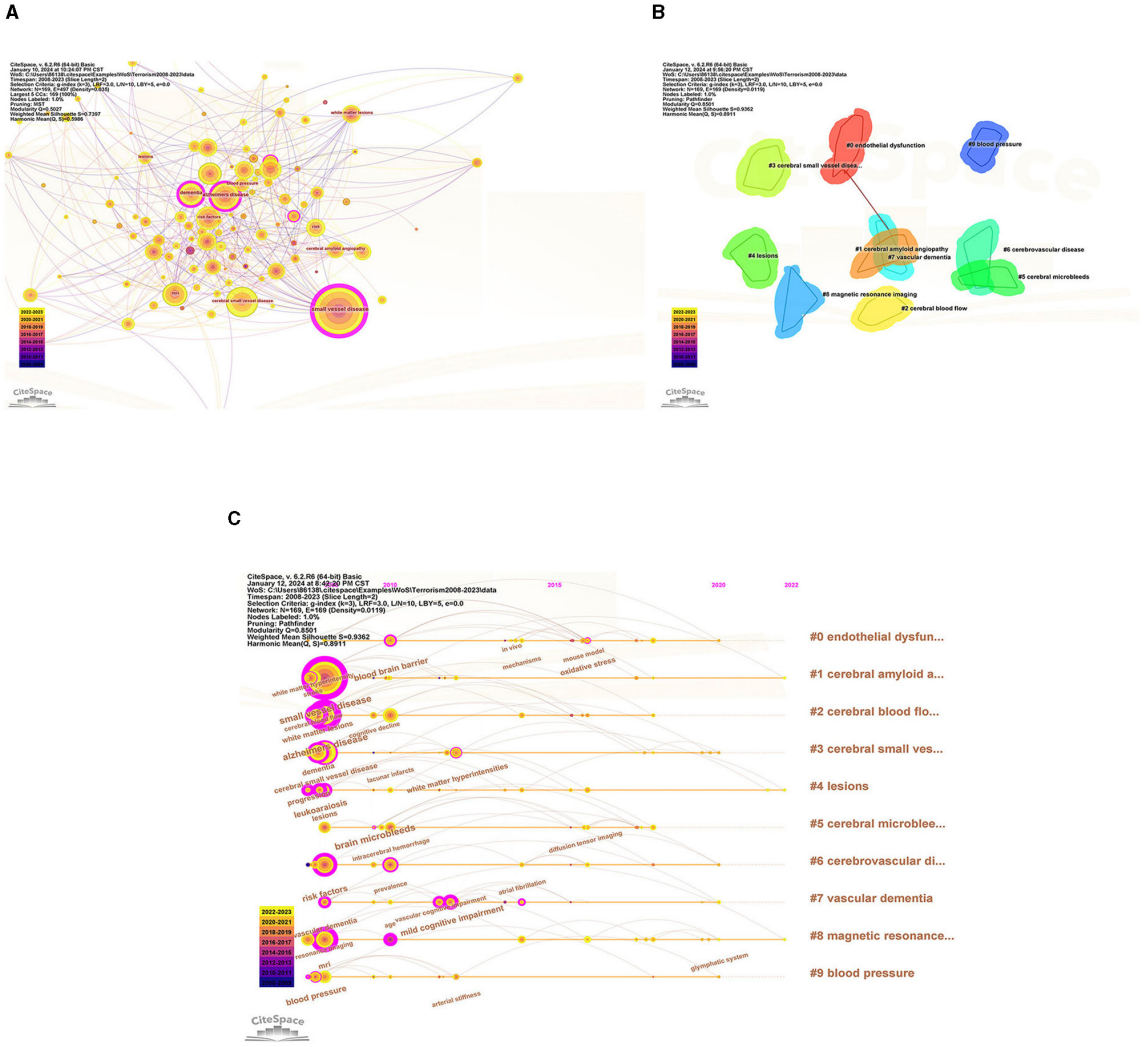
**(A)** The network map of keywords. **(B)** Clustered network of keywords. **(C)** The timeline view of keywords.

To identify popular topics and assist scholars to better understand current scientific concerns, cluster analysis could be used to cluster the included keywords. Keyword co-occurrence and network cluster analysis are both available through CiteSpace. The hot spots of CSVD research are illustrated in Table 5 and Figure 8A, which display the most commonly used keywords. The most relevant terms in CSVD research were identified by the keyword co-occurrence clusters using the hierarchical cluster labeling method; the cluster modularity *Q* value is 0.8501, while the mean contour value *S* value is 0.9326. Typically, a *Q* value > 0.3 represents a significant clustering structure; if the average cluster contour value *S* > 0.5, the clustering is generally considered reasonable. If the *S*-value > 0.7, the clustering result is considered convincing. This study identified 10 clusters of keywords, which includes “endothelial dysfunction” (cluster #1), “cerebral amyloid angiopathy” (cluster #2), “cerebral blood flow” (cluster #3), “cerebral small vessel disease” (cluster #4), “lesions” (cluster #5), “cerebral microbleeds” (cluster #6), “cerebrovascular disease” (cluster #7), “vascular dementia” (cluster #8), “magnetic resonance imaging” (cluster #9), and “blood pressure” (cluster #10) (Figure 8B). To depict the development of high-frequency keywords within each cluster, CiteSpace developed a keywords timeline viewer that could cluster keywords and take time into consideration. The viewer might also make it straightforward to identify the time frame for a specific subject and the development of this research area. Each stage and evolution path of the CSVD research's concentration could be intuitively understood, as shown in Figure 8C. This study formed 10 clusters of keywords, showcasing the knowledge structure and dynamic changes in the CVSD field to some extent. Table 6 contains a list of clusters in summary form.

**Table 5:** The top 20 keywords associated with CSVD research.

**Rank**	**Keyword**	**Count**	**Rank**	**Keyword**	**Count**
1	Small vessel disease	1,370	11	Cognitive impairment	466
2	Stroke	1,058	12	White-matter hyperintensity	461
3	Cerebral small vessel disease	907	13	Magnetic resonance imaging	429
4	Dementia	868	14	Cerebral amyloid angiopathy	412
5	MRI	748	15	White-matter lesions	375
6	Alzheimer's-disease	657	16	Cerebral microbleeds	363
7	Small-vessel disease	566	17	Microbleeds	358
8	Risk-factors	565	18	Leukoaraiosis	354
9	Brain	559	19	Prevalence	342
10	Risk	514	20	Ischemic-stroke	329

**Table 6:** Keyword cluster analysis.

**Cluster ID**	**Size**	**Silhouette**	**Mean (year)**	**Top terms (LSI)**
0	17	1	2014	Endothelial dysfunction; oxidative stress; blood brain barrier; microglia; magnetic resonance imaging
1	16	1	2011	Cerebral amyloid angiopathy; small vessel disease; white matter hyperintensity; cerebral small vessel disease; Alzheimer's disease
2	14	0.985	2011	Cerebral blood flow; Alzheimer's disease; cognitive impairment; white matter lesions; ischemic stroke
3	14	1	2013	Cerebral small vessel disease; white matter hyperintensities; white matter; lacunes; lacunar infarcts
4	13	0.921	2013	Lesions; progression; leukoaraiosis; dysfunction; infarcts
5	13	0.937	2014	Cerebral microbleeds; intracerebral hemorrhage; amyloid angiopathy; cortical superficial siderosis; cerebral amyloid angiopathy
6	13	0.976	2011	Cerebrovascular disease; risk factors; atrial fibrillation; prevalence; cerebral infarction
7	13	0.885	2013	Vascular dementia; vascular cognitive impairment; mild cognitive impairment; intracerebral hemorrhage; blood-brain barrier
8	12	0.931	2016	Magnetic resonance imaging; cerebral small vessel diseases; cognitive dysfunction; glymphatic system; perivascular spaces
9	12	1	2011	Blood pressure; arterial stiffness; lacunar stroke; risk; pulse-wave velocity

**Figure 9:**
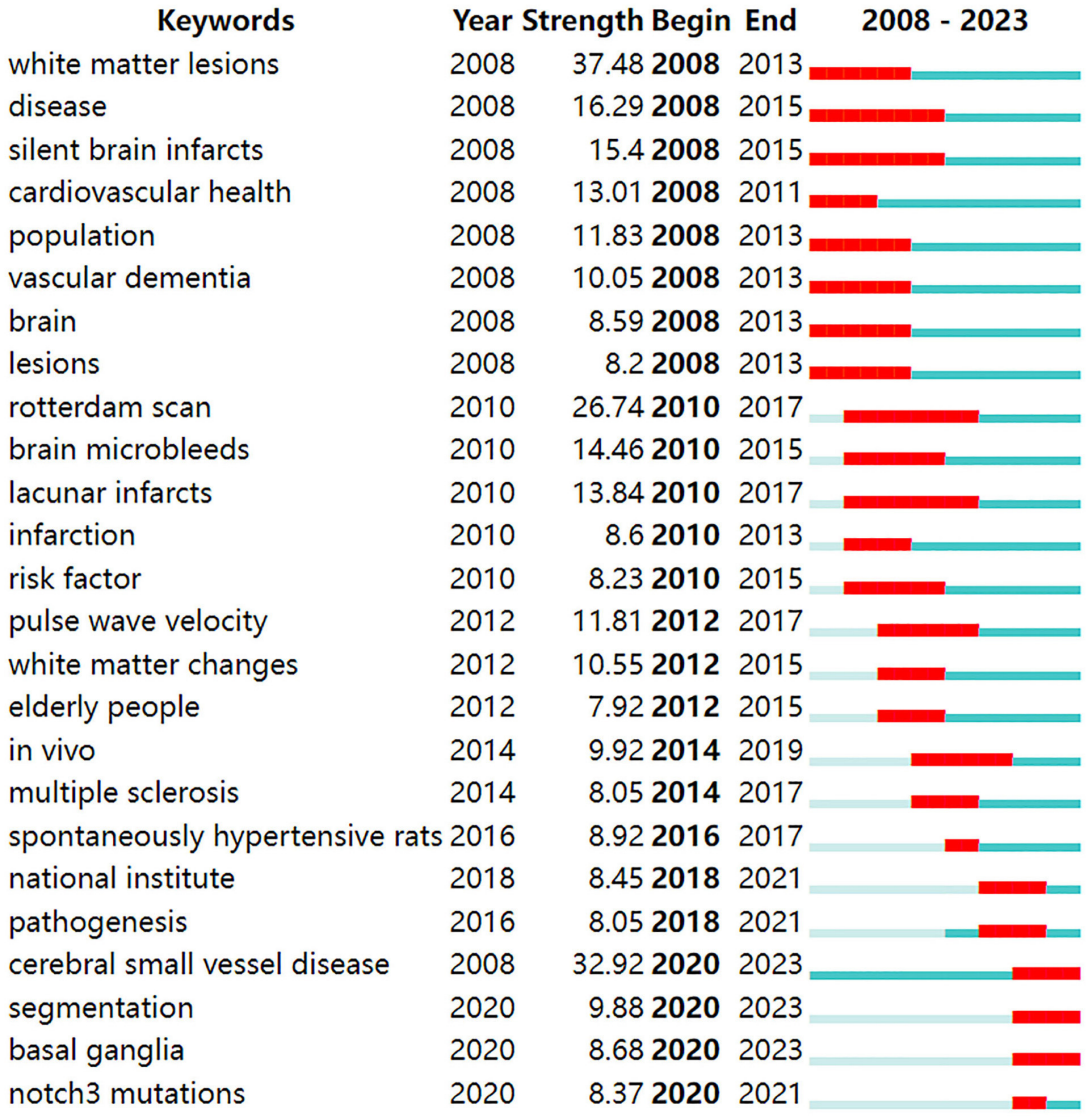
Top 25 keywords with the strongest citation bursts.

According to the detection of emerging words with high frequency and fast growth rate within a period of time, keywords with high-burst intensity are an important indicator reflecting research hot spots, frontiers, and latest trends (Figure 9). Twenty-five new words were identified, with the term “white matter lesions” having the highest increase in citations at 37.48 in 2008. The keyword bursts among of them that lasted until 2023 included “cerebral small vessel disease,” “segmentation,” and “basal ganglia,” which represented the hot spots in recent years.

## 4 Discussion

### 4.1 General information

This study examined the characteristics of articles in the CSVD field over the last 15 years to describe the current research and help guide future research. The number of publications per year was tracked to identify trends over the past 15 years. Highly cited articles, defined as those in the top percentile of citations, were noted as these tend to signify groundbreaking research. Productivity of countries was quantified by the number of CSVD publications across the time period. Specific research institutions and individual authors with extensive CSVD-focused portfolios were documented as leading producers. Using keyword analysis, prevalent research themes and hot topics were identified. Mapping these bibliometric parameters over time illustrated the evolution of global CSVD research. As shown in Figure 2, only 88 articles were published in 2008, suggesting that researchers were just beginning to understand CSVD. Since then, there has been a steady increase in the number of research papers focusing on CSVD, particularly in the past couple of years. This trend suggests that the study of CSVD is gaining more research interest and continues to be a popular research topic. The future trajectory remains positive. However, the number of mean citations is not smooth. The later the papers are published, the lower their times of being cited. The higher the mean citations, the more important the publication in the field. The mean citations peaked in 2013, suggesting that this was a pivotal year during which influential literature may have been published. Wardlaw et al. (2013a) paper titled “Neuroimaging standards for studying small vessel disease and its impact on aging and neurodegeneration” achieved the highest citation, and supported the correlation between the peaks in mean citations and the publication of seminal literature.

The analysis of countries showed that the USA made the highest contribution in terms of NP and NC, indicating that it is a top-performing and influential country in the field of CVSD research, with strong collaboration networks with China and England. Although the NP of China ranked second, the numbers of NC and AC were relatively low. These data suggest that China is experiencing a discrepancy between the number and standard of its publications. To tackle this problem, it is important to improve cooperation with various nations, particularly the United States, The Netherlands, and Germany, while also closely monitoring advancements in science and conducting thorough research to enhance the quality of publications. At the institutional level, the University of Edinburgh published the most articles in this field.

Wardlaw Joanna M., a researcher at the University of Edinburgh's Research Center for Stroke and Dementia, holds the record for the highest number of publications in this particular field of study. Among the top 10 most productive institutions, universities make up the main body, of which 40% were from The Netherlands and accounted for the largest proportion. In China, Capital Medical University and Fudan University are the most productive institutions in the CVSD field. In terms of research institutions, strengthening the cooperation between different institutions or teams is extremely important for future basic or clinical trials of CVSD. Wardlaw, Joanna turned out to be the most productive author in the CVSD field among the top 10 authors with high output. It is important to mention that Wardlaw is one of the top 10 authors frequently cited together, focusing on various articles related to the mechanisms, clinical importance, and neuroimaging criteria for small vessel disease (Arba et al., 2017; Francis et al., 2019; Horsburgh et al., 2018; Wardlaw et al., 2013a, 2019, 2013b). Furthermore, He participated in many clinical trials of CSVD (Blair et al., 2022; Markus et al., 2022) and was involved in the development of guidelines to provide evidence-based recommendations to assist with clinical decisions about CSVD management (Wardlaw et al., 2021). In this ESO Guideline on covert CVSD, he recommended patients with CSVD and hypertension to have their blood pressure well-controlled, as lower blood pressure targets may reduce CSVD progression; furthermore, he does not recommend antiplatelet drugs such as aspirin in CSVD. While the AC of Dishpans, martin was ranked first, but the gap is very subtle. Dishpans, Martin is a neurologist from the German Center for Neurodegenerative Disease. He is famous for his contributions to investigate the impact of lesion location in processing speed in age-related small vessel disease (Agirman et al., 2021; Duering et al., 2014). The VOSviewer software automatically categorized authors into distinct groups. The authors from the same clusters contributed excellent scientific works to their region. We also observed Charidimou, Andreas as a seconded TLS (Figure 5), whose research focused on cerebral amyloidosis and cerebral hemorrhage associated with CVSD (Castello et al., 2021; Charidimou et al., 2019; Scheumann et al., 2020). Wardlaw, Joanna was a bridge connection with other authors. The Boston criteria version 2.0 was established for the evaluation of cerebral amyloid angiopathy in a multicenter and retrospective study (Charidimou et al., 2022). Collaboration has always been an important requirement for the advancement of scientific discovery.

Among the top 10 journals with the highest productivity, *Stroke, Neurology*, and *Frontiers in Neurology* stood out as the most productive, specializing in CSVD. The number of publications shows an increasing trend year by year, suggesting that CSVD is a relatively new and in-demand research field. In addition, the *Stroke* journal has gradually widened the gap with other journals, which likely indicates that research in the CSVD field is deepening and expanding, and academic collaboration may become more and more important to solve complex problems and improve the quality of research. More significant research findings are anticipated to be released in *Stroke* in the coming years, leading to an increase in both impact factor and scientific merit. Scientists must monitor this journal to track the advancement of CSVD and anticipate future developments. Moreover, analyzing the journal could assist researchers in expediting the research process by promptly determining the most suitable journals for submission. As shown in Figure 6C, the presence of these citation paths also highlights the importance of knowledge transfer across disciplines. Researchers in different fields often refer to and build upon each other's work, leading to the advancement and development of new ideas and approaches in various scientific domains. Overall, these identified citation paths provide insights into the interconnections of different fields of study, research trends, potential collaboration opportunities, and knowledge transfer across disciplines.

Analyzing co-citations is a useful way to evaluate the degree of connection among articles. In the professional field, the importance of an article is believed to increase with the frequency of its citations (Wu et al., 2021). Most of the top 10 cited references were published in top-ranked journals, and three articles were written by Wardlaw et al. (2013a,b, 2019); this result also confirmed the results of the author analysis. In 2013, Wardlaw, Joanna established the imaging criteria for CSVD for the first time, which have been referenced since then. Four out of the top 10 cited references focused on the pathogenesis and clinical characteristics of CSVD. Understanding the pathology and clinical manifestations of disease can help people better recognize the disease, prevent it, treat it, and improve their quality of life. Three out of the top 10 references cited focused on the correlation between cognitive dysfunction and CSVD. The article titled “Blood-Brain Barrier: From Physiology to Disease and Back,” focused on BBB transport physiology, endothelial and pericyte transporters, and perivascular and Para vascular transport. Additionally, it examines rare genetic neurological disorders that originate from defects in BBB-related cells, highlighting the connection between BBB dysfunction and neurodegeneration (Sweeney et al., 2019). Based on this, Wardlaw et al. found emerging targets for new therapies including brain barrier integrity, vascular reactivity, vascular compliance, perivascular inflammation, and myelin repair (Jian et al., 2020).

Citation bursts are citations that have been heavily cited by other researchers recently, indicating new trends in a particular area of study. The initial reference burst originated in 2010 because of studies that were published in 2014. Greenberg et al. (2009) have suggested a step-by-step manual for detecting CMBs and recommend potential future strategies for understanding the significance of these frequent abnormalities as indicators of, and contributors to, small-vessel brain disorders. Chabriat et al. (2009) summarized the knowledge of CADASIL (Cerebral Autosomal Dominant Arteriopathy with Subcortical Infarcts and Leukoencephalopathy)—the primary genetic factor leading to stroke and vascular dementia in adults. Both the above articles were published in *Lancet Neurol*. The strongest burst reference was the international neuroimaging standards for CSVD (Wardlaw et al., 2013a). There were nine article bursts from 2020 up to the present time (Bernal et al., 2020; Cannistraro et al., 2019; Cuadrado-Godia et al., 2018; Liu X. et al., 2018; Low et al., 2019; Ma et al., 2019; Rensma et al., 2018; Ter Telgte et al., 2018; Wardlaw et al., 2019), and the successful publication of these articles clarify the mechanism, pathology, and clinical manifestations of CSVD. Among them, new papers reporting vascular inflammation and endothelial dysfunction may be the driving force behind CSVD; however, the associations between systemic inflammation and CSVD remain inconclusive.

Keywords summarize the main concepts of an article and are typically seen as crucial markers that indicate the research focus and trending topics within a particular field (Yi et al., 2018). Analyzing the top 20 most frequent keywords reveals that “small vessel disease” and “Alzheimer's disease” were among them. In terms of different forms of coexisting disease in CSVD, Alzheimer's disease (AD) and small vessel disease often coexist (Gocke et al., 1970). Alzheimer's disease shares common risk factors such as high blood pressure and diabetes, and pathophysiological processes like oxidative stress, inflammation, mitochondrial dysfunction, and metabolic abnormalities with CSVD. Damage to the wall can cause microaneurysms to grow externally as a result of fibrosis and narrowing or blockage of the nearby lumen (Liu Y. et al., 2018). Pathologically, wall damage can lead to external expansion of microaneurysms due to fibrosis and proximal luminal stenosis or obstruction (Chou et al., 2021). Ultimately, dysfunction in the regulation of the affected small blood vessels causes a reduction in blood flow to the brain and long-term inadequate blood supply to the brain, while blockage of the artery results in sudden lack of blood flow, resulting in lacunar infarction (Tang and Liu, 2021). Severe stenosis and hypoperfusion involving multiple arterioles, mainly deep white matter, leading to incomplete ischemia, are seen on neuroimaging as “white matter hyperintensity” (WMH) (Wang et al., 2021). From the beginning, genetics have played a role in influencing the risk of developing AD. Early-onset AD has been linked to familial autosomal-dominant genes *PSEN1, PSEN2*, and the amyloid precursor protein (*APP*) gene (Lamb, 1997). Furthermore, multiple genetic indicators can influence both AD and CVSD. It is uncertain if these genes contribute to the development of CSVD and subsequently impact AD, or if they directly impact both CSVD and AD. The ε4 variant of the apolipoprotein E (*APOE*) gene increases the risk of both AD and CSVD (Kim et al., 2020).

The evolution and change of keywords over time reflect, to some extent, the development of hot spots and can guide future research directions. The research core themes can be distilled into the following characteristics by using four analyses: #2, # 7, and # 9 are disease research fields, mainly related to the sequel caused by CSVD, including keywords such as dementia, cognitive impairment, and lacunar stroke; #0 and #1 are pathological changes of CSVD, mainly including endothelial dysfunction, oxidative stress, and cerebral amyloid angiopathy; #3, #4, #5, #6, and #8 are the markers on magnetic resonance imaging (MRI) and influence changes caused by CSVD, including leukoaraiosis, white matter, cerebral micro bleeds, cardiovascular spaces, and MRI.

Burst detection, which utilizes algorithms to identify significant changes in events, can be executed in CiteSpace. There are two characteristics associated with the burst: its strength and duration. Trending search terms have increased in popularity over time, indicating the level of interest from people. “Cerebral small vessel disease” lasted from 2008 until 2023, which means the people pay more attention to it. The keyword bursts among them that lasted from 2020 to 2023 included “cerebral small vessel disease,” “segmentation,” and “basal ganglia,” which have represented the hot spots in recent years.

### 4.2 Research core themes

#### 4.2.1 Clinical manifestation of CSVD

CSVD can present with different symptoms based on the underlying cause of the condition and the areas of the brain that are impacted. Individuals may present sudden onset stroke symptoms, progressive cognitive deterioration, dementia, gait disorder, sphincter dysfunctions, and psychiatric disorders (Del Bene et al., 2013; van der Flier et al., 2005; Rensma et al., 2020). Cognitive decline caused by CSVD presents with executive dysfunction, attention and memory decline, set-shifting disabilities, slower speed of information processing, decline of verbal fluency, and delayed recall. Symptoms of apathy, mood disorders, depression, and difficulties with daily activities were observed in the behavioral domain. Currently, the cause of cognitive impairment in CSVD is still unknown. It has been suggested that cognitive dysfunction in CSVD patients may be related to atherosclerosis and microvascular dysfunction (Rensma et al., 2020), which may affect cerebral blood perfusion, neurogenesis, and brain self-regulation, resulting in both neurological and cognitive dysfunction in CSVD patients (Rensma et al., 2020).

Additionally, lacunar infarctions make up 25% of total acute ischemic strokes. Although lacunar strokes may occasionally result from mechanisms of brain ischemia, such as cardiac embolism or carotid artery stenosis, most result from intrinsic diseases of the small deep perforating arteries (Bailey et al., 2012). Hypertension and diabetes mellitus are commonly linked to lacunar stroke (Wardlaw, 2005). The SPS3 trial (Secondary Prevention of Small Subcortical Strokes) is the largest study to date that enrolled cases of MRI confirmed lacunar stroke (Benavente et al., 2012). The SPS3 trial tested two randomized interventions: clopidogrel and aspirin vs. aspirin alone and two target levels of systolic blood pressure (Benavente et al., 2012). Dual antiplatelet therapy increased the incidence of major hemorrhage and mortality rate, and thus, the trial was stopped early. Furthermore, the risk of recurrent stroke was not significantly reduced by dual antiplatelet therapy. The SPS3 was also designed to test whether a systolic blood pressure target < 130 mmHg compared with 130–149 mmHg would be associated with a reduction of all strokes (ischemic and hemorrhagic). Although there was a 19% decrease in all strokes that was not statistically significant, the authors found a significant decrease in intracerebral hemorrhage (Benavente et al., 2013). Recent research indicates that maintaining strict control over blood pressure may be advantageous for individuals who have experienced a lacunar stroke. Currently, antiplatelet monotherapy is recommended to prevent recurrent strokes after lacunar strokes, because dual antiplatelet therapy might increase major bleeding risk without providing additional stroke-reduction benefits. A recent trial called PICASSO (Prevention of Cardiovascular Events in Asian Patients with Ischemic Stroke at High Risk of Cerebral Hemorrhage) evaluated the efficacy and safety of cilostazol vs. aspirin. Cilostazol was found to be just as effective as aspirin in preventing cardiovascular events in the study; however, it did not lower the occurrence chance of a hemorrhagic stroke (Kim et al., 2018). A recent randomized controlled study found that individuals with minor ischemic stroke or high-risk transient ischemic attack had a reduced risk of major ischemic events but an increased risk of major hemorrhage at 90 days when treated with both clopidogrel and aspirin, compared to those treated with aspirin alone (Johnston et al., 2018).

#### 4.2.2 The pathological changes of CSVD

The exact pathophysiological processes of CSVD remain unknown. We observed that the solid line of cluster#0 named “endothelial dysfunction” was initially believed to be an etiological contributor to CSVD, which halted in recent years on the timeline view. A previous study indicated that endothelial dysfunction is the key initiator for CSVD and its pathogenesis, predating BBB breakdown (Karlsson et al., 2012). Endothelial cells (ECs) act as a barrier between tissue and blood, controlling blood flow, managing transport of circulating components, and playing a role in inflammatory processes. Endothelial dysfunction-induced brain damage manifests in several ways *via* different mechanisms. The ECs control the local CBF by secreting vasodilatory mediators (NO and prostacyclin) or vasoconstrictors (endothelin) in response to chemical or mechanical stimulators which constitute the endothelial-dependent vasodilatory or vasoconstrictor response (Iadecola and Gottesman, 2019). The endothelial cells are also involved in creating the blood-brain barrier (BBB) by forming tight junctions (Iadecola and Gottesman, 2019). The reduction in NO synthesis in EC dysfunction can also lead to VCAM-1 expression, which is an adhesion molecule that is induced by proinflammatory cytokines (Liao, 2013).

“Oxidative stress” become a hot spot in 2006. Oxidative stress is a result of oxidant overproduction caused by NADPH oxidases, malfunction of antioxidant enzymes or decreased activity of these enzymes. The relationship between oxidative stress and endothelial dysfunction is bidirectional and interconnected: (1) Oxidative stress can directly damage endothelial cells, leading to dysfunction. Overproduction of ROS can lead to oxidative changes in lipids, proteins, and DNA in endothelial cells, disrupting their usual operation; and (2) Endothelial dysfunction can also contribute to oxidative stress. A dysfunctional endothelium produces fewer amounts of protective molecules like nitric oxide, which normally help counteract oxidative stress. This imbalance can further increase ROS production and exacerbate oxidative damage. As mentioned, hypertension and aging are considered the most important risk factors for CSVD. Some of the key features of CSVD have been described in mouse models of hypertension including reductions in CBF; impaired vasodilator capacity (e.g., NO-dependent responses, neurovascular coupling, and autoregulation); inward remodeling; and BBB disruption (De Silva and Miller, 2016). Angiotensin II, the primary active peptide in the renin-angiotensin system, seems to have a significant impact on these vascular irregularities, leading to an elevation in superoxide generation by Nox2-NADPH oxidase in cerebral vessels of rodents (De Silva and Miller, 2016).

Over the course of 15 years, “cerebral amyloid angiopathy” remained a focal point of ongoing interest and significance. Cerebral amyloid angiopathy is defined by the deposition of amyloid β (Aβ) in the walls of small arteries, arterioles, and capillaries of the leptomeninges, cerebral cortex, and cerebellar cortex, and it is the dominant cause of lobar intracerebral hemorrhage. Aβ protein accumulation in capillaries affects BBB integrity, which leads to a loss of tight junction proteins and increased BBB permeability (Holland et al., 2008). Then, perivascular edema and extravasation of toxic plasma components caused by the disruption of BBB contributes to localized damage to brain parenchyma and EPVS (Hartz et al., 2012). Cerebral amyloid angiopathy also plays a role in causing cognitive decline as a separate symptom. The cause of cognitive decline related to cerebral amyloid angiopathy remains unknown; numerous research studies have suggested that cognitive decline and dementia may be attributed to diffuse brain microbleeds, micro-infarcts, hypoperfusion, and white matter hypoxia resulting from vessel changes linked to CAA, distinct from AD and Lewy body pathology (Shi and Wardlaw, 2016; Zhang et al., 2017).

#### 4.2.3 CSVD markers on MRI and the influence changes caused by CSVD

The MRI features of CSVD mainly include recent small subcortical infarcts, lacunes, WMHs, EPVS, CMBs, and brain atrophy. Small vessel disease lesions are considered permanent, with WMHs representing demyelination, axon loss, and gliosis, lacunes being cavities replacing destroyed tissue, and microbleeds being fixed hemorrhages. Multiple longitudinal studies indicate progression of WMHs, with only few reported instances of slight reductions in WMH volume (Schmidt et al., 2016). Bilateral, mostly symmetrical hyperintensities on T2-weighted and fluid-attenuated inversion recovery (FLAIR) MRI are characteristic features of white matter lesions accompanied with some hypointense difference from the cerebrospinal fluid (CSF) on T1-weighted MRI and low density on cerebral CT in most older individuals with or without cognitive decline. In addition to white matter, the hyperintense lesions are also located in subcortical gray matter structures, such as the basal ganglia and brainstem (Wardlaw et al., 2013b). The inclusion of hyperintensities in gray matter and brainstem as part of WMH is a topic of debate (Schmidt et al., 2011; Wardlaw et al., 2013b). Diffusion tensor (DT)-MRI and magnetization transfer (MT)-MRI were utilized more frequently to distinguish WMH from other types of brain lesions like lacuna and atrophy, to investigate their individual effect on dementia by providing quantitative data on the condition of the brain's white matter. CMBs are MR-visible small (generally 2–5 mm in diameter, but up to 10 mm) areas of signal void caused by perivascular collections of hemosiderin deposits that are foci of past hemorrhages resulting from small vessels involved in CAA or arteriolosclerosis (Shi and Wardlaw, 2016). Perivascular spaces (PVS), as also known as Virchow–Robin spaces, are extensions of the extracerebral fluid-filled spaces that follow the typical course of a vessel as it goes through gray or white matter (Aribisala et al., 2013). EPVS are predominantly located in the basal ganglia and show increased signal intensity equal to CSF on T2-weighted images, appear hypointense on T1-weighted and occasionally hypointense on FLAIR images without a hyperintense rim to be distinguished from old lacunar infarcts (Aribisala et al., 2013).

### 4.3 Research hotpots

#### 4.3.1 MRI segmentation

Magnetic resonance imaging (MRI) segmentation is a process of dividing an MRI scan into different regions or segments based on the underlying anatomical characteristics. Segmentation is essential in a variety of medical uses, including diagnosing diseases, planning treatments, and monitoring progress. MRI segmentation aims to partition an image into meaningful regions that represent different tissues or structures such as organs, tumors, or blood vessels. This process can be performed manually by experts, but it is time-consuming and subjective. Therefore, automated or semi-automated techniques have been developed to assist in MRI segmentation. The neuroimaging features of CSVD include recent small subcortical infarct (RSSI), WMH, lacune, PVS, CMB, and brain atrophy. In recent years, samples of segmentation or detection have been made to automatically quantify the MRI manifestations of CSVD for better efficiency and reproducibility in research or clinical settings, and/or to associate these MRI features with possible clinical consequences for a better understanding of CSVD.

Clinically apparent RSSI, also called lacunar stroke, is defined as recent infarction (within the past few weeks) in the area supplied by a single perforating artery (Wardlaw et al., 2013b) that can be identified on MRI diffusion-weighted imaging (DWI) sequence as hyperintense lesions of up to 20 mm in diameter on axial sections. Early automated segmentation techniques for acute lesions on DWI typically relied on basic characteristics (such as intensity and edge details) that were not sufficiently reliable because of the large variations in lesion patterns (Prakash et al., 2006). With the increased application of deep-learning methods in recent studies, such as deep convolutional neural networks, high-level features of lesion patterns can be extracted that have achieved better performance than the traditional methods. The technique obtained a Dice similarity coefficient (DSC) of 79.13% and a lesion-wise precision, showing spatial agreement between the automatic segmentation results and the ground truth (Zhao et al., 2020). WMHs can be seen as bright spots on FLAIR and T2-weighted (T2w) MRI scans. Many approaches have been recently proposed for automatic segmentation of WMHs. Indeed, the performances of different WMH segmentation methods are generally not fully comparable because of the difference of subject characteristics and lesion load across studies. For example, the methods that achieved DSC of >0.80 were generally evaluated in stroke patients or patients with vascular dementia (Wardlaw et al., 2013a), where the patients tend to have larger lesion burden of WMH. In this regard, independent evaluations for different level of lesion load should be encouraged for a better generalizability of the segmentation performance. Lacunes can be seen as round or ovoid subcortical fluid-filled cavities in MRI with diameters of ~3–15 mm. Lacunes present as hypointensities in T1-weighted (T1w) and FLAIR images and hyperintensities in T2w images (approximate to the intensity of CSF). Few studies have suggested automated approaches for identifying lacunes, typically relying on intensity-based algorithms. CMBs can be identified as small (up to 10 mm) areas of signal void on MRI sequences such as T2*-weighted gradient-recalled echo (GRE) or susceptibility-weighted images (SWI), and the SWI was reported to have better reliability and SE for CMB detection than T2*GRE (Cheng et al., 2013). Using automated detection techniques can reduce the burden on neuroradiologists and enhance the accuracy and speed of CMB identification. Supervised CMB detection methods using convolutional neural network (CNN) have advanced owing to the progress in deep-learning techniques. In a study with the largest benchmark data set available for CMB detection on SWI, Qi et al. (2016) applied 3D CNN and achieved an SE of 93.16%, precision of 44.31%, and FP/CMB of 1.17. When using 7T SWI instead of 1.5T/3T SWI, slightly better SE, FP/CMB, and much higher precision could be achieved. Automatic quantification methods for EPVS are significantly less developed than WMH and CMB. Dubost et al. (2019) developed an automated rating method of EPVS based on 3D regression fully CNN on 1.5T T2w image. Park et al. (2016) used manually delineated PVS masks as the ground truth for learning based on 7T MRI. Evaluation of brain shrinkage can be conducted in specific cerebral lobes, particular tissues, or distinct regions of the brain. In-depth evaluation of regional brain shrinkage typically involves segmenting brain tissues and specific structures using automated segmentation techniques, with statistical parametric mapping (SPM) being a widely used method for this purpose. Additionally, the methods include atlas-based (based on the accurate alignment of atlas priors), learning-based (based on an annotated training set), and algorithmic methods (relies on intensity information to a greater extent, e.g., region-based and deformable methods). Different methods may have their strengths and limitations for the segmentation of specific structures. In conclusion, MRI segmentation will be a research hot spot in the future of CSVD, and is currently undergoing rapid evolution to identify the best segmentation model for the imaging manifestations of CSVD. This approach could become a hot spot for future work and provide a stronger support for enhancing the diagnostic accuracy of CSVD and offer inspiration for exploring treatment targets.

#### 4.3.2 Basal ganglia EPVS

CSVD is characterized by a range of markers that are clearly defined such as lacunes, WMHs, and CMBs. Currently, more attention is given to other markers such as EPVS. Vascular stiffness, inflammation, protein deposits, and disruption of the BBB—partly due to CSVD—decreases the clearance of solutes from the brain interstitial fluid (Brown et al., 2018). EPVS have been linked to various conditions such as head trauma, cerebrovascular accident, and idiopathic normal pressure hydrocephalus. EPVS are frequently found in the centrum semiovale and basal ganglia. Previous research has shown that age, high blood pressure, and WMHs are linked to an increased risk of basal ganglia enlarged perivascular spaces (BG-EPVS), indicating a potential connection to hypertensive arteriopathy (Charidimou et al., 2013). The potential mechanisms have been proposed. Increased intraluminal pressure may cause greater extravasation of fluid through the small arteries into PVS, which is supported by rat experiments in which sustained hypertension could cause increased permeability of endothelial cells and fluid-induced damage to the surrounding brain tissue (Gutierrez et al., 2013). Additionally, increased pulpability in these regions may result in the expansion of PVS due to their nearness to brain tissue (Gutierrez et al., 2013). However, the consequences of BG-EPVS are generally considered clinically silent; a few ongoing research studies have investigated the link between BG-EPVS and cognitive impairments (Hernández et al., 2013; Zhu et al., 2010). Nevertheless, the findings varied among the different research projects. The reason behind the connection between BG-EPVS and cognitive decline is still not fully understood, and it is speculated whether the connection is because of the direct impact of EPVS or the presence of CSVD markers such as WMH, CMB, and lacunes. Over a period of 5 years, Huijts et al. (2013) examined 189 individuals at high risk for cerebrovascular disease and discovered a correlation between the rise in BG-EPVS and a decline in cognitive processing speed. Interestingly, this connection was independent of age and high signal intensity in the white matter. However, Arba et al. (2018) found that the number of BG-EPVS was not associated with cognitive impairment. Additionally, recent research has shown a connection between BG-EPVS and gait disturbances (Yang et al., 2022). The exact pathophysiological processes responsible for the link between BG-EPVS and walking remain unclear. We hypothesized that an abundance of BG-EPVS could interfere with the operation of the basal ganglia, brain network, and brain environment, potentially resulting in difficulties with walking and balance. As mentioned above in the analysis, the prevalence of MRI segmentation has increased in recent years. Further work is needed to address the issue of lesion type discrimination and develop methods for quantifying disease markers in order to analyze accuracy results by disease load and inform a better the scope of their applicability.

## 5 Limitations

This study has some limitations. First, citation analysis favors English language journals, so our findings emphasize Western research. Manual screening of abstracts was not feasible which may have resulted in the inclusion of some irrelevant articles. Second, while WoSCC has a wider scope than databases like Scopus, PubMed, Cochrane, and Embase Library databases, it still has a bias toward English-language journals. A prior analysis found that over 95% of sources indexed in WoSCC publish in English. This potentially excluded relevant non-English publications and contributed to geographic biases, potentially only highlighting Western authors. Expanding the database sources could provide more diversity in the literature reviewed. Third, reliance on citation analyses introduces biases toward older, more established research areas and academics. Recently published innovative research is less likely to be highly cited initially compared to seminal work that has accumulated citations for decades. Finally, bibliometric software such as CiteSpace and VOSviewer cannot provide statistical functions, so it is impossible to understand the actual situation of publications in different countries.

Overall, while bibliometric analyses allow for broad mapping of research fields, they have limitations regarding representativeness. Combining database sources, expanding citation and publication timeframes, and implementing manual full-text reviews could reduce biases, but likely at the expense of capturing a narrower slice of literature. Researchers must balance the strengths and limitations of these approaches when aiming to review and summarize evidence.

## 6 Conclusion

We analyzed the research progress, hot spots, and frontiers in this field; studied CSVD through bibliometric analysis; and revealed the future research prospects. Currently, research on CSVD is in a rapid development stage, and since 2008, publications related to CSVD have steadily increased. We also identified leading countries, institutions, and leading scholars in the field and analyzed journals and representative literature. Keyword co-occurrence analysis and burst graph emergence detection have identified “MRI segmentation” and “Basal ganglia enlarged perivascular spaces” as the most recent areas of research interest. The cause of CSVD remains unknown and requires additional investigation. Although this study utilized the most extensive database available, it is possible that it overlooked studies that were not included in the database. Subsequent evaluations could potentially explore various databases. In conclusion, bibliometric analysis provides objective insights into the research of CSVD and addresses further research opportunities and challenges.

## Data Availability

The original contributions presented in the study are included in the article/supplementary material, further inquiries can be directed to the corresponding authors.
